# Preventive Effects of Thinned Apple Extracts on TNF-α-Induced Intestinal Tight Junction Dysfunction in Caco-2 Cells through Myosin Light Chain Kinase Suppression

**DOI:** 10.3390/foods11121714

**Published:** 2022-06-11

**Authors:** Joo-Yeon Lee, Choon Young Kim

**Affiliations:** Department of Food and Nutrition, Yeungnam University, Gyeongsan 38541, Korea; jooyeonlee@ynu.ac.kr

**Keywords:** thinned apple, tight junction, myosin light chain kinase, intestinal epithelial barrier, inflammatory bowel disease

## Abstract

Inflammatory bowel disease (IBD) is associated with intestinal epithelial barrier dysfunction and elevation of proinflammatory cytokines such as TNF-α. Tight junctions (TJ) control the paracellular barrier of the gut. Thinned apples are an indispensable horticultural agro-waste for apple cultivation, but are disposed by most farmers. This study aimed to elucidate the preventive effect of thinned apple extracts (TAE) on the intestinal epithelial barrier dysfunction induced by TNF-α treatment in Caco-2 cells. The differentiated Caco-2 monolayers were pre-treated with mature apple extract (MAE) and TAE for 1 h and then incubated with 100 ng/mL TNF-α for 24 h. The TJ integrity was estimated by measuring the value of transepithelial electrical resistance (TEER) and the flux of fluorescein isothiocyanate-dextran through paracellular transport. TAE had a better protective effect on the intestinal epithelial barrier than MAE did. Western blot results showed that TAE pre-retreatment elevated TJ protein levels such as claudin-1, -4, and -5. Moreover, TAE inhibited the interaction between zonula occludens proteins (ZO)-1 and occludin by reducing the tyrosine phosphorylation of ZO-1. The mechanisms underlying TAE-mediated attenuation of TNF-α-induced TJ disruption included suppression of myosin light chain kinase and NF-κB p65 protein levels. Therefore, thinned apples could be a sustainable ingredient for functional foods to prevent IBD.

## 1. Introduction

The valorization of agro-waste has recently been an important environmental and economic research topic [1]. Agro-wastes, such as crop residue, weeds, forest waste, and livestock waste, are inevitably generated during the agricultural production processes [2]. These wastes cause environmental and economic problems because they are generated in large amounts annually and are usually discarded [2]. Due to the presence of abundant phenolic compounds and their toxic potential, incineration of agro-wastes causes environmental deterioration [2]. From an economic perspective, agro-wastes are costly to dispose of [1]. Moreover, they contain abundant organic and bioactive molecules, and thus require intensive efforts to develop strategies for using them as value-added products. Importantly, agro-waste has received worldwide attention for various applications, for example, to utilize as a fertilizer, to obtain compounds such as dietary fibers, proteins, and phytochemicals, and to produce biofuel [1]. Therefore, the valorization of agro-wastes without disposal contributes to sustainable economic and environmental development.

Thinned apples, one of the surplus agro-wastes produced in apple cultivation, are known to possess potent health benefits [3]. They are cultivated and then removed in the post-bloom period to evenly spread nourishment in the orchard, while many are discarded annually as waste [4]. Several studies have found that thinned apples contain approximately 10 times higher polyphenol compounds than mature apples do [5]. In particular, procyanidins, epicatechin, chlorogenic acid, and phloridzin are known to be the main polyphenols within them [3]. Due to their high polyphenol content, thinned apples have various physiological functions, including anti-cancer, anti-obese, anti-cardiovascular, and anti-inhibitory activities [5,6]. Therefore, thinned apples are potential bioactive materials for the development of functional foods that contribute to sustainable development.

The tight junction (TJ), a network of proteins connecting adjacent intestinal cells, plays a pivotal role in controlling the intestinal epithelial barrier function [7]. TJs are composed of multiple proteins, such as zonula occludens proteins-1 (ZO-1), ZO-2, ZO-3, occludins, claudins, and cingulin [8]. TJs help maintaining cell polarity by separating the apical and basolateral regions in the small intestine, and regulate paracellular permeability to block large harmful molecules, microbiomes, and toxins [7,8]. Paracellular permeability regulation by TJ proteins is governed by ZO-1 phosphorylation and the interaction between phosphorylated ZO-1 and occludin in Caco-2 cell monolayers [9].

TJ disruption is critical in the pathogenesis of gastrointestinal diseases, such as inflammatory bowel disease (IBD), colorectal cancer, and irritable bowel syndrome [10]. Tumor necrosis factor-alpha (TNF-α), a pro-inflammatory cytokine, is locally and systemically high in patients with intestinal diseases and is known to increase inflammatory responses and TJ permeability [8,10]. Thus, TNF-α leads to cytoskeletal deterioration by translocating TJ proteins from the plasma membrane to the cytosol and by decreasing TJ protein levels [11]. TNF-α controls integrity by regulating the interaction between occludin and ZO-1 proteins, protein expression, phosphorylation, and reorganization [12]. Furthermore, nuclear factor kappa-B (NF-κB) pathway activation is induced by TNF-α elevates myosin light chain (MLC) kinase (MLCK), leading to MLC phosphorylation [13]. Subsequently, the phosphorylated MLC contracts the actomycin ring to increase intestinal permeability and results in the endocytosis of TJ proteins. Therefore, the maintenance of TJ proteins by MLCK pathway inhibition is a key molecular target for preventing intestinal diseases.

Various polyphenols have been reported to possess beneficial activities against stimuli-induced TJ alterations [9]. When Caco-2 cells stimulated by interferon-gamma and TNF-α were treated with berberine, the main component of *Coptidis rhizome*, it attenuated pro-inflammatory cytokines and disrupted TJ integrity through occludin redistribution [14]. Kaempferol, found in various fruits and vegetables, has been shown to reduce methyl-β-cyclodextrin-mediated TJ dysfunction through upregulation of ZO-1, -2, occludin, claudin-1, -3, and -4 protein expression and occludin phosphorylation [15]. Additionally, quercetin, a potent antioxidant found in red onions and kale, promoted TJ integrity, as shown by increased transepithelial electrical resistance (TEER) values and decreased paracellular permeability in a dose-dependent manner. Quercetin at 100 µM concentration increased claudin-4 protein levels and fortified the assembly of ZO-2, occludin, and claudin-1 by suppressing PKCδ [16]. Although thinned apple contains approximately 10 times more polyphenols than mature apple, few limited studies have been conducted to evaluate its role in intestinal barrier function. Thus, this study aimed to investigate the preventive effect of thinned apple extracts (TAE) on TNF-α-induced TJ dysfunction in an in vitro cell culture model, the Caco-2 monolayer.

## 2. Materials and Methods

### 2.1. Reagents

Thinned and mature apples were obtained from an orchard located in Gyeongsan, Gyeongsangbuk-do, Korea. To estimate the integrity and paracellular permeability of intestinal junctions, transwell plates were purchased from Corning (New York, NY, USA). Fluorescein isothiocyanate-dextran (FITC-dextran, MW 4000 Da) and all other chemicals were purchased from Sigma-Aldrich Co. (St. Louis, MO, USA). Cell culture media (Dulbecco’s modified Eagle’s medium, DMEM), trypsin, fetal bovine serum (FBS), phosphate-buffered saline (PBS), and related reagents were purchased from Welgene (Daegu, Korea) and Gibco (Grand Island, NY, USA). Claudin-1, claudin-4, claudin-5, ZO-1, and occludin primary antibodies were purchased from Santa Cruz Biotechnology Inc. (Dallas, TX, USA). Horseradish peroxidase-conjugated secondary antibodies were purchased from Jackson ImmunoResearch Laboratories (West Grove, PA, USA).

### 2.2. Sample Preparation

Thinned apples and mature apples were obtained from an apple orchard located in Kyungsan, Korea. All apples (Malus pumila Mill., Gyeongsan, Gyeongbuk, Korea) were washed, freeze-dried, and ground. Each ground sample was added to distilled water (1:10, *w*/*v*) and extracted by autoclaving (VS-1221, Vision, Seoul, Korea) at 121 °C for 20 min. The solutions were filtered through a cheesecloth. The filtrates were centrifuged at 1500× *g* for 20 min, and the supernatants were lyophilized. The final extract was frozen until the assay.

### 2.3. Cell Culture and Treatment

Caco-2 cells (passage 4–17) were incubated at 37 °C in a 5% CO_2_ atmosphere. Caco-2 cells were grown in DMEM with 4500 mg/L D-glucose, L-glutamine, 110 mg/L sodium pyruvate, 1200 mg/mL sodium bicarbonate, 100 units/mL penicillin, 100 µg/mL streptomycin, and 10% FBS. Caco-2 cells were seeded on the membrane (apical compartment) in 12-transwell plates. The apical compartment was filled with 0.5 mL culture medium and the basolateral compartment contained 1.5 mL medium. All cell experiments were performed after 14 days, yielding a TEER of 250 Ω × cm^2^. The thinned and mature apple extracts were apically pre-treated with 100 ng/mL TNF-α in the apical compartment for 1 h and then in both compartments for 24 h [12].

### 2.4. Determination of Transepithelial Electrical Resistance and Paracellular Permeability

A Millicell ERS-2 resistance meter was used to measure the TEER of Caco-2 monolayers, as previously described [12]. Each TEER was calculated by multiplying the resistance (Ω) and the membrane area (cm^2^). The effect of Caco-2 monolayer paracellular permeability was determined using an FITC-dextran marker (4 kDa). The flux of FITC-dextran was determined as previously described [12]. Fluorescence was measured using a fluorescent plate reader (Molecular Devices, Chicago, IL, USA) with an excitation wavelength of 485 nm and an emission wavelength of 535 nm. This was calculated using the following equation, compared to the control group.

(1) $$\begin{array}{c} \mathrm{Paracellular}\;\mathrm{permeability}\;\left(\%\;\mathrm{of}\;\mathrm{control}\right)\\ =\frac{\mathrm{fluorescence}\;\mathrm{of}\;\mathrm{medium}\;\mathrm{in}\;\mathrm{treatment}–\mathrm{fluorescence}\;\mathrm{of}\;\mathrm{initial}\;\mathrm{medium}}{\mathrm{fluorescence}\;\mathrm{of}\;\mathrm{medium}\;\mathrm{in}\;\mathrm{non}-\mathrm{treatment}–\mathrm{fluorescence}\;\mathrm{of}\;\mathrm{initial}\;\mathrm{medium}}\text{×}\;100 \end{array}$$

### 2.5. RNA Isolation and Reverse Transcription

Filter-grown Caco-2 cells were washed with ice-cold PBS. Total Caco-2 RNA was isolated using TRIzol (Invitrogen, Carlsbad, CA, USA). Total RNA concentration was determined by measuring the absorbance at 260/280 nm. Total RNA was reverse-transcribed to cDNA using the iScript™ cDNA Synthesis Kit (Bio-Rad Laboratories, Hercules, CA, USA). The total reaction volume was 20 µL and was prepared by adding 5X iScript Reaction Mix 4 μL, iScript Reverse Transcriptase 1 μL, total RNA, and RNase-water. Reverse transcription reactions were performed in a Simpli Amp Thermal Cycler (Applied Biosystems, Waltham, MA, USA) at 25 °C for 5 min, 46 °C for 20 min, and 95 °C for 1 min.

### 2.6. Gene Expression by Quantitative RT-PCR Analysis

Quantitative real-time PCR analysis was conducted using the StepOnePlus Real-Time PCR System (Applied Biosystems, Waltham, MA, USA) and SYBR Green (2X SYBR Green Master Mix, Applied Biosystems, Foster city, CA, USA) according to the manufacturer’s instructions. The experiments were performed in duplicate for each sample, using the 2^−ΔΔCt^ analysis method. The expression levels of target genes were normalized to that of the reference gene, 18S rRNA. The primer sequences used for RT-qPCR were designed with Primer Express software 3.0.1 (Applied Biosystems, Waltham, MA, USA) and are listed in Table 1 [12].

### 2.7. Preparation of Cell Lysate and Immunoblot Analysis 

Caco-2 cells were lysed with cell lysis buffer (100 mM Tris-HCl, 100 mM NaCl, 0.5% Triton-X, 1 mM sodium orthovanadate, and 10 mM sodium fluoride) containing a protease inhibitor cocktail (GenDEPOT, Barker, TX, USA). Cell lysates were centrifuged at 13,000× *g* for 10 min at 4 °C. The supernatant of the cell lysates was run on an SDS-polyacrylamide gel and electroblotted onto a polyvinylidene difluoride membrane. The membranes were incubated successively with antibodies against claudin-1, -4, and -5, occludin, ZO-1, MLCK, NF-κB, and β-actin, and then with a horseradish peroxidase-conjugated anti-rabbit or anti-mouse IgG antibody. Reactive bands were detected using the Chemiluminescent Western Blot Imaging System (300, Azure Biosystems, Dublin, CA, USA), and signals were visualized using ImageJ [14].

### 2.8. Immunoprecipitation 

The phosphorylation and interaction of TJ proteins were analyzed by immunoprecipitation. Caco-2 cell lysates were obtained by treating the appropriate experimental reagents for varying experimental periods. The supernatant of cell lysates containing the Protein A/G PLUS-Agarose beads (Sigma-Aldrich Co., sc-2003) was precleaned at 4 °C for 30 min and then centrifuged at 40× *g* for 5 min to transfer the supernatant to a new tube. ZO-1 antibody and supernatant of the cell lysate mixture were incubated for 12 h at 4 °C. Protein A/G PLUS-Agarose beads were then added to the mixture and incubated for 2 h at 4 °C. Subsequently, the tubes containing the immunoprecipitates were centrifuged, and the supernatants were aspirated. The pellets were then washed five times with PBS. Gel loading buffer (2X Laemmli sample buffer) was then added to the pellets. The samples were boiled for 10 min at 100 °C, after which ZO-1 proteins were detected by western blot analysis [15].

### 2.9. Polyphenol Contents by High-Performance Liquid Chromatography

The phenolic content of the samples was analyzed by high-performance liquid chromatography (HPLC). Each sample was passed through PVDF membrane filters with a 0.45 µm pore size (Millipore, Billeria, MA, USA). Next, 10 µL of each filtered sample was injected into an HPLC device (Waters 2695, Waters Co., Milford, MA, USA) equipped with a UV detector (Waters 2489, Waters Co.) and Atlantis dC18 column (4.6 mm × 150 mm, 5 µm, Waters Co.) operated at 34 °C. The phenolic content was analyzed at 280 nm. The mobile phase consisted of 1% phosphoric acid (solvent A) and 100% acetonitrile (solvent B) at a 1.0 mL/min flow rate. Gradient elution was performed as follows: 0 min, 90% solvent A, 10% solvent B; 0–27 min, 60% solvent A, 40% solvent B; 28–55 min, 56% solvent A, 44% solvent B; 56–60 min, 90% solvent A, 10% solvent B. Peak identification was performed according to standards (catechin, chlorogenic acid, epicatechin, phloridzin, and procyanidin).

### 2.10. Statistics

Statistical significance of differences between mean values was assessed using Duncan’s multiple range test, Dunnett’s *t*-test and Student’s *t*-test. All reported significance levels represent *p*-values. Statistical significance was set at *p* < 0.05. Each experiment was performed in duplicate or triplicate (*n* = 2 or 3), and all experiments were repeated at least three times to ensure reproducibility. The SPSS 25.0 software was used for statistical analysis.

## 3. Results

### 3.1. The Thinned Apple Extract Is Superior to Mature Apple Extract in the Prevention of TNF-α-Mediated Deterioration of TJ Integrity

**Figure 1:**
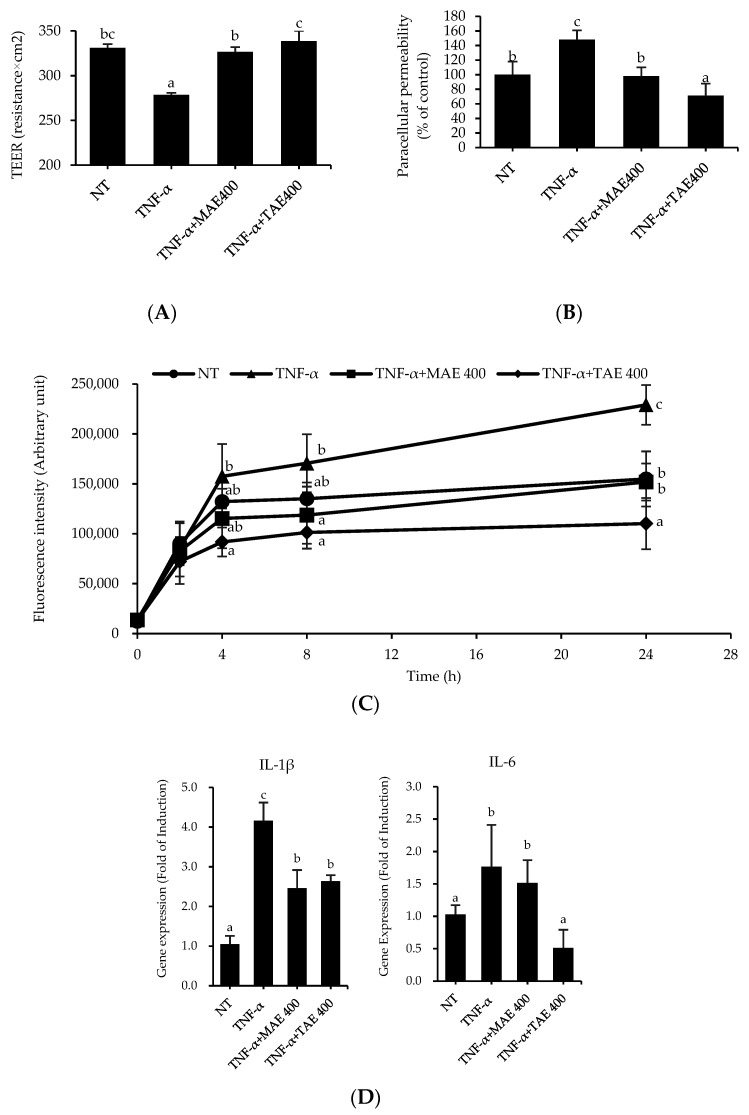

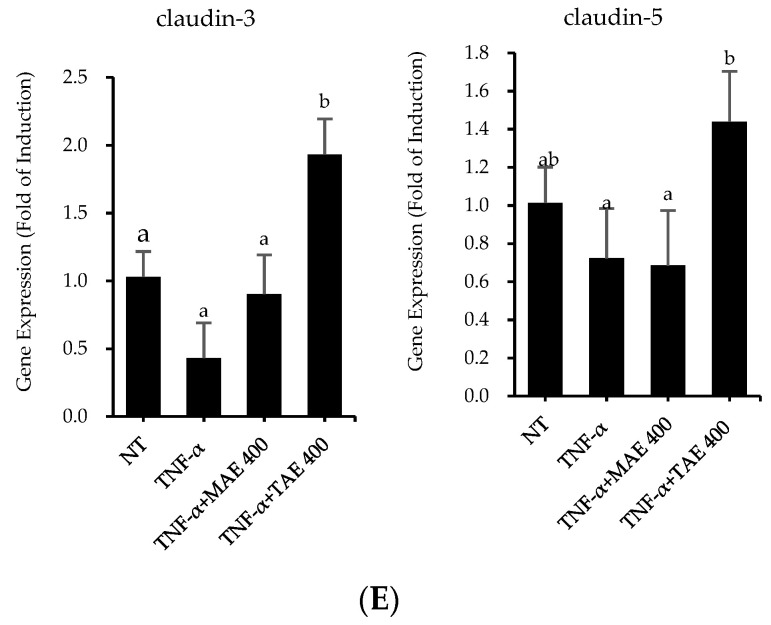
Caco-2 monolayers were apically pre-treated with mature apple extract (MAE) and thinned apple extract (TAE) for 1 h in the apical compartment and then incubated with 100 ng/mL of tumor necrosis factor-alpha (TNF-α) in both apical and basolateral compartments for 24 h. Tight junction integrity was determined by (**A**) the transepithelial electrical resistance (TEER) value and (**B**) paracellular permeability at 24 h. (**C**) The time course of paracellular permeability was tested in TNF-α treated with/without MAE and TAE. Gene expression levels were estimated by quantitative RT-PCR analysis of (**D**) interleukin-beta (IL-1β) and IL-6, and (**E**) claudin-3 and claudin-5. 18S gene expression was measured as a loading control. Results are shown as mean ± SEM (*n* = 3–6). Different letters indicate significant differences by Duncan’s multiple range test (*p* < 0.05).

To compare the effect of TAE with mature apple extract (MAE) on TJ integrity, TEER and paracellular permeability were assessed. Caco-2 monolayers were pre-treated with MAE or TAE for 1 h in the apical compartment and then incubated with 100 ng/mL TNF-α for 24 h in the apical and basolateral compartments. As shown in Figure 1A, TNF-α treatment reduced the TEER value by 0.84 times compared to that of the non-treated (NT) group. However, the MAE and TAE pre-treatment groups significantly inhibited the TNF-α-mediated reduction of the TEER value. Consistent with this, the paracellular permeability demonstrated by the flux of 4 kDa FITC-dextran in the TNF-α-treated group was 47% higher than that in the NT group (Figure 1B). The flux of 4 kDa FITC-dextran in MAE- and TAE-treated groups was significantly lower than that in the TNF-α-treated group, indicating that both prevented the reduction of TJ integrity induced by TNF-α. A time-course examination of paracellular permeability revealed that the flux of 4 kDa FITC-dextran in Caco-2 monolayers of all groups increased rapidly up to 4 h and then slowly up to 24 h (Figure 1C). Both the MAE- and TAE-treated groups tended to have lower paracellular permeability than that of the TNF-α-treated group. However, the paracellular permeability of the TAE-treated group was superior to that of the MAE-treated group. After 4 h, significant differences between the NT and TAE groups were observed for each measurement. Since TNF-α treatment reduces TJ protein levels and increases inflammation, gene expression levels of proinflammatory cytokines, enzymes, and claudin were investigated by real-time PCR analysis [17]. As shown in Figure 1D, the transcript levels of IL-1β and IL-6 in the TNF-α-treated group increased 3.96- and 1.71-fold, respectively, compared to those of the NT group. By contrast, MAE treatment lowered the gene expression levels of IL-1β and IL-6 by 1.69- and 1.16-fold, respectively, compared to those of the TNF-α-treated group. TAE treatment similarly suppressed the TNF-α-mediated increase in gene expression levels of IL-1β and IL-6 by 1.58- and 1.16-fold, respectively, compared to those of the NT group. However, the gene expression differences between the MAE- and TAE-treated groups were not statistically significant, except for the IL-6 gene. The expression level of claudin, an important transmembrane TJ protein, was evaluated in TNF-α-treated Caco-2 monolayers following MAE or TAE pre-treatment (Figure 1E). Compared to the NT group, claudin-3 and -5 in the TNF-α-treated group exhibited 2.39- and 1.40-fold reduction in gene expression, respectively. Moreover, MAE treatment slightly reversed the TNF-α-mediated reduction of claudin-3 and -5 expression; however, the difference was not statistically significant. Interestingly, TAE significantly increased the claudin-3 and -5 expression levels by 4.48- and 1.99-fold, respectively, in the TNF-α-treatment group. TAE pre-treatment increased the gene expression of TJ proteins. These data suggest that TAE is capable of attenuating TNF-α induced TJ dysfunction in vitro. This is consistent with previously reported TEER and paracellular permeability experiment results. Thus, if the small intestinal epithelial barrier is loosened due to TNF-α, TJ proteins affect the mobility and integrity of TJs quantitatively and morphologically [18].

### 3.2. Quantification of Polyphenolic Compounds Present in the Mature and Thinned Apple Extracts by HPLC Analysis

Since TAE was more effective than MAE in preventing intestinal epithelial barrier dysfunction in Caco-2 monolayers, differences in the composition and content of polyphenol between TAE and MAE were analyzed by HPLC (Table 2). As expected, all polyphenol contents were higher in TAE than in MAE. The polyphenol composition of TAE was in the following descending order: chlorogenic acid, epicatechin, catechin, phloridzin, and procyanidin B1. Chlorogenic acid was the most abundant phenolic compound in TAE and MAE. The amounts of chlorogenic acid, epicatechin, catechin, phloridzin, and procyanidin B1 in TAE were 6172.03, 2027.13, 1646.39, 1081.61, and 792.32 μg/g, respectively.

### 3.3. Thinned Apple Extracts Prevent TNF-α-Mediated TJ Alteration by Regulating TJ Protein Expression

**Figure 2:**
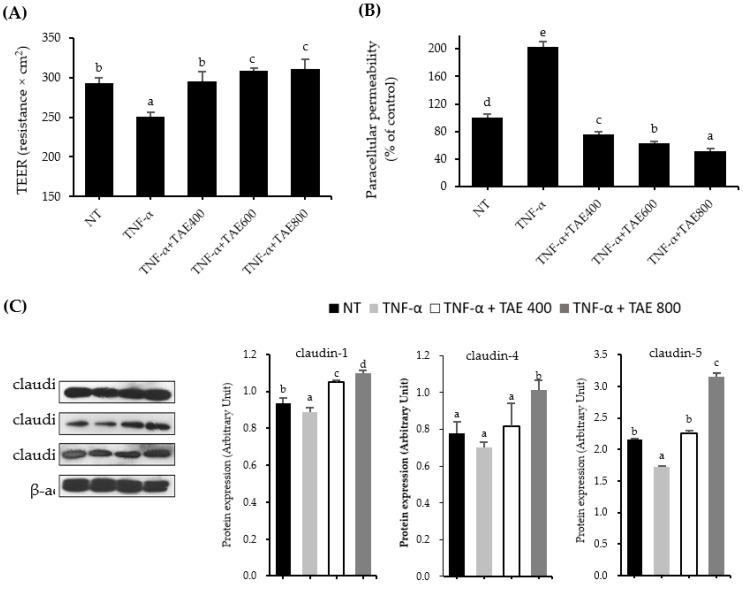

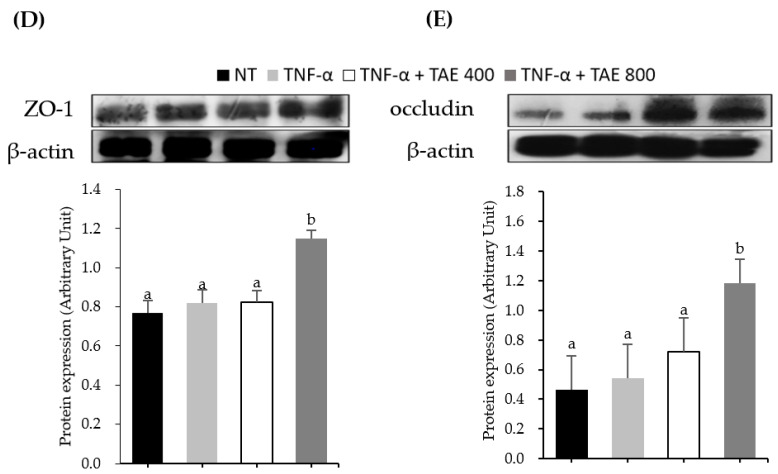
Preventive effects of thinned apple extract (TAE) on tumor necrosis factor-alpha (TNF-α) reduced tight junction integrity in Caco-2 cells. (**A**) Transepithelial electrical resistance (TEER) and (**B**) paracellular permeability were measured in TNF-α-treated Caco-2 cells at 24 h incubation. The protective effect of TAE on the protein expression levels of (**C**) claudin-1, claudin-4, claudin-5, (**D**) ZO-1, and (**E**) occludin. Cells were treated with TAE for 1 h and then treated with 100 ng/mL of TNF-α for 24 h. Data are expressed as mean ± SD (*n* = 6–9). Different letters indicate significant differences by Duncan’s multiple range test (*p* < 0.05).

Since the preventive effect of TAE on TNF-α-attenuated TJ function was more potent than that of MAE, the effect of TAE on TJ integrity was scrutinized. Caco-2 monolayers were pre-treated with different TAE concentrations (400, 600, and 800 µg/mL) for 1 h and then incubated with 100 ng/mL TNF-α for 24 h. As illustrated in Figure 2A and B, TAE dose-dependently inhibited the influence of TNF-α on TJ integrity. TEER values of TAE-treated cells at concentrations of 400, 600, and 800 µg/mL followed by TNF-α treatment were 295, 309, and 311 Ω·cm^2^, respectively, whereas that of TNF-α-treated cells treated was 251 Ω·cm^2^. Consistent with the TEER values, paracellular permeability in the TNF-α-treated group significantly increased approximately two-fold; however, TAE treatment abolished TNF-α action.

When the effect of TAE on TJ protein levels was determined by western blot analysis, TAE at 800 µg/mL exhibited significantly increased protein levels of claudin-1, -4, -5, ZO-1, and occludin (Figure 2C). TAE at 800 µg/mL increased TJ protein expression of claudin-1, -4, and -5 by 1.23-, 1.44- and 1.83-fold, respectively, as compared with that of the TNF-α treated group (Figure 2C). Although ZO-1 and occludin protein expressions were not significantly altered by TNF-α treatment in Caco-2 monolayers, TAE at 800 µg/mL increased ZO-1 and occludin protein expression levels by 1.4- and 2.17-fold, respectively, compared to those of the TNF-α-treated group (Figure 2D,E). Therefore, TAE maintained and/or enhanced TJ protein expression in TNF-α-treated Caco-2 monolayers.

### 3.4. Thinned Apple Extract Prevents TJ Disruption by Modulating the Interaction between ZO-1-Occludin and Myosin Light Chain Kinase Protein

**Figure 3:**
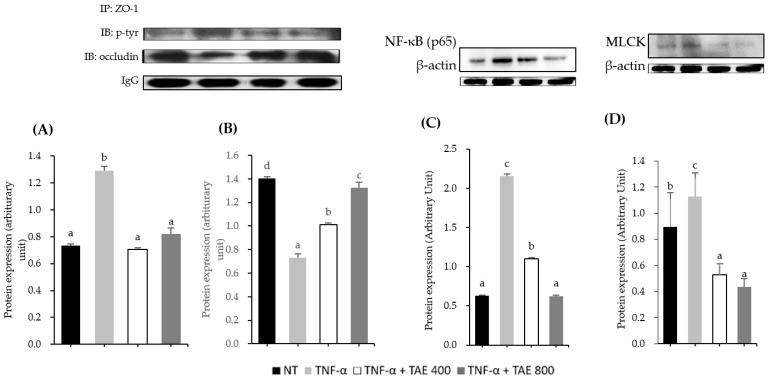
Effect of thinned apple extract (TAE) on tyrosine phosphorylation of ZO-1 and the interaction between the ZO-1 andoccludin in Caco-2 cells. (**A**) The protein level of tyrosine phosphorylation (*p*-tyr) and (**B**) occludin that immunoprecipitated with ZO-1. The protein level of (**C**) p65 and (**D**) myosin light chain kinase (MLCK) in Caco-2 cells. Data are expressed as mean ± SD (*n* = 3–6). Different letters indicate significant differences according to Duncan’s multiple range test (*p* < 0.05).

Although TNF-α did not affect ZO-1 and occludin protein levels (Figure 3), they were regulated by its phosphorylation and protein–protein interaction. Thus, we confirmed the effect of TAE on the phosphorylation and interaction of ZO-1 and occludin. TAE at a concentration of 800 µg/mL reduced tyrosine phosphorylation of ZO-1 1.58-fold, compared to that of the TNF-α treatment group (Figure 3A). As a result of the immunoprecipitation assay used to study the interaction between ZO-1 and occludin, the dissociation of ZO-1 with occludin was 1.92-fold in the TNF-α-treated group compared to that in the NT group. However, TAE treatment at 800 µg/mL revealed a 1.81-fold increase in the occludin-ZO-1 complex compared to that of the TNF-α-treated group (Figure 3B). These results indicate that TAE augments TJ integrity, which was reduced by TNF-α-induced ZO-1 protein phosphorylation and the interaction between ZO-1 and occludin. 

TNF-α has been reported to decompose TJ protein expression by increasing MLCK protein through NF-κB pathway activation [19]. The p65 protein, a subunit of NF-κB, was upregulated by TNF-α, while TAE at a concentration of 800 μg/mL reduced p65 protein expression 3.45-fold (Figure 3C). Through the activation of the NF-κB pathway, TNF-α significantly increased MLCK protein expression levels, but was suppressed approximately 2.58-fold by TAE treatment.

## 4. Discussion

Agro-waste generated from agricultural practices is rich in various bioactive compounds and can be utilized to contribute to sustainable development [1]. Thinned apple, an indispensable horticultural agro-waste of the apple cultivation process, contains more polyphenols than mature apples [5]. Meanwhile, tight junctions (TJs) consisting of multi-protein networks between small intestinal cells are a major factor in determining epithelial barrier function [7]. Gastrointestinal diseases, such as inflammatory bowel disease (IBD), colorectal cancer, and irritable bowel syndrome, and other metabolic diseases, such as obesity and diabetes, are closely related to altered TJ function [8]. Pathogenic microorganisms, proinflammatory cytokines, food allergies, and stress are the major stimuli that worsen TJ integrity by activating inflammatory mechanisms. While various polyphenols have been demonstrated to improve the symptoms of certain diseases by reinforcing TJ integrity, the role of thinned apple in the intestinal epithelium remains elusive. Thus, this study aimed to investigate the beneficial effects of thinned apple extract on TNF-α-induced TJ dysfunction in Caco-2 monolayers. 

Since the health benefits of mature apples are well known, their effects on TJ integrity were compared with those of thinned apples. When TJ integrity was determined by transepithelial electrical resistance (TEER) and flux of FITC-dextran in Caco-2 monolayers, Thinned apple extract (TAE) pre-treatment alleviated TNF-α-induced TJ dysfunction more than that of mature apple extract (MAE) (Figure 1). Previous studies reported that apple extracts or nutrients enhance intestinal TJ integrity [20]. Treatment of the digested apple extract decomposed by pepsin, pancreatic fluid, and bile salt increased the TEER value of Caco-2 cells two-fold compared to that of the control [20]. Moreover, apple treatment protected against disrupted TJ function in Caco-2 cells incubated with stimuli. TJ alteration in Caco-2 cells treated with free radical oxidant was suppressed by various concentrations (10–100 µg/mL) of apple extract as evidenced by higher TEER values than that of the control [21]. Another study showed that treatment of Caco-2 cells monolayers with polyphenol extracts from apple peel reversed both 3.2 mM p-cresol-induced decrease in TEER value and increased flux of FITC-dextran [22]. Furthermore, an in vivo study supports the beneficial effect of apple polysaccharide on intestinal barrier function. Rats fed a high-fat diet for 10 weeks developed obesity and intestinal dysfunction, while body weight change and intestinal permeability were significantly reduced [23]. Together with these studies, our study data supported the fact that thinned apples, which have more polyphenols than mature apples, strengthen the intestinal epithelial barrier.

Gastrointestinal diseases are characterized by a high level of inflammatory responses due to local and systemic pro-inflammatory cytokines, and these cytokines exacerbate the symptoms of diseases. Therefore, the suppression of the inflammatory cytokine production is one of the important pathophysiological targets to treat and/or ameliorate the symptom of gastrointestinal diseases. MAE and TAE downregulated gene expression of interleukin (IL)-1β and IL-6 compared to TNF-α treated group (Figure 1D), indicating that both have anti-inflammatory activity. Based on Figure 1, both TAE abolished TNF-α-stimulated TJ dysfunction and inflammation, supporting that TAE fortifies intestinal function. Similarly, polyphenols in thinned apples exhibited anti-inflammatory activities both in vitro and in vivo. Lauren et al. demonstrated that thinned apple polyphenols inhibit lipopolysaccharide (LPS)-induced the gene expression of TNF-α in RAW264.7 macrophage cells [24]. Yoshioka et al. reported that thinned apple procyanidins suppressed IFN-γ synthesis in intraepithelial lymphocytes and inhibited phorbol 12-myristate 13-acetate-induced expression of IL-8 in intestinal epithelial cells [25]. The oral administration of thinned apple polyphenols attenuated gene expression of IFN-γ, TNF-α, IL-1β, IL-6, IL-17, and IL-22 in the colons of mice with colitis [26]. Thus, it seems that polyphenols in TAE have the potential to alleviate inflammation. 

In order to establish why TAE is superior to MAE, the polyphenol contents of chlorogenic acid, epicatechin, catechin, phloridzin, and procyanidin B1 were studied. As shown in Table 2, TAE has a significantly higher polyphenol content than that of MAE (Table 2). Zheng et al. reported that the polyphenol content and antioxidant activities in apple decreased rapidly after the 85th day of thinning (size about 5 cm diameter). Consistent with this, as the apples grew, antioxidant capacity estimated by oxygen radical absorbing capacity, 2,2-diphenyl-1(2,4,6-trinitropheyl)hydrazine-1yl (DPPH) radical scavenging activity, and ferric reducing antioxidant power assays decreased rapidly in the early cultivation stage and then slightly reduced later [27]. TAE in this study were about 2 cm in diameter, which have been known to have a variety of biological activities because of their high polyphenol contents. 

According to Table 2, chlorogenic acid is the most abundant in TAE. Numerous in vivo and in vitro studies have demonstrated that chlorogenic acid exhibits health-promoting activities including antioxidant and anti-inflammatory benefits. Zhang et al. evaluated chlorogenic acid on colon damage in a C57BL/6J mice model of dextran sulfate sodium (DSS)-induced colitis. Chlorogenic acid reduced the disease activity index and TNF-α levels in colon tissues. Additionally, it ameliorated DSS-induced inflammatory responses, reduced colon shortening, and decreased the histological scores [28].

A randomized, double-blind study by Akazome et al. demonstrated that intake of ApplePhenon^®^, polyphenols derived from immature green apples within 8–12 weeks, decreased visceral fat accumulation [29]. Another study reported that dietary apple polyphenol extracts from unripe apples suppressed proliferation, metastasis, and cancerous hypercholesterolemia in Donryu rats implanted with AH109A cells [30]. Taken together, these results suggest that the polyphenols in TAE are responsible for the preventive action of TJ function exacerbated by TNF-α. 

Since TAE has higher TJ protective activity than that of MAE, the role of TAE was more closely investigated. First, TAE prevented TNF-α-induced alteration of TJ in a dose-dependent manner based on TEER and paracellular permeability values (Figure 2A,B). The data revealed that the TAE group increased TJ protein expressions of zonula occludens (ZO)-1, occludin, claudin-1, -4, and -5, which were inhibited by TNF-α treatment (Figure 2C–E). In particular, Günzel et al. reported that claudin proteins play an important role in passing substances through the lumen of the small intestine [17]. Barmeyer et al. reported that downregulation of claudin-4, -5, and -8 led to epithelial barrier dysfunction in HT-29 cells treated with TNF-α and IFN-γ [31]. These previous studies support our results that indicate that increasing claudin protein expression by TAE aids TJ function. In our study, the protein expression levels of occludin and ZO-1 were increased by TAE. However, discrepant results showed no changes in occludin and ZO-1. Even though TEER values were increased, berberine treatment at 100 µM for 48 h did not influence the protein expressions of ZO-1 and occludin in Caco-2 cells treated with or without 10 ng/mL TNF-α and 10 ng/mL IFN-γ [13]. Similarly, improvements in TJ integrity in Caco-2 cells treated with both 10 ng/mL TNF-α and 10 µM rebeccamycin for 24 h was observed; however, ZO-1 and occludin protein levels remained unchanged [32]. Although certain studies supported that occludin and ZO-1 protein expressions do not essentially affect TJ function, it is believed that this discrepancy might be due to the concentration of TNF-α treatment. Zhang et al. used the same concentration of TNF-α as in this study and showed that TNF-α treatment at 100 ng/mL significantly suppressed occludin protein expression; however, co-treatment with propofol enhanced occludin expression [33].

Occludin and ZO-1 proteins play a pivotal role in TJ regulation by phosphorylation and migration, as well as interactions between occludin and ZO-1 proteins [34]. Thus, further investigation was performed to clarify whether reorganization and post-translational modification of occludin, and ZO-1 proteins were regulated by TAE treatment, which ameliorated ZO-1 phosphorylation and increased the interaction with ZO-1 and the occludin complex (Figure 3). In line with our results, increased tyrosine phosphorylation of ZO-1 and decreased interaction between occludin and ZO-1 through deposition of claudin-4 were associated with fungal toxin-induced TJ function alteration in Caco-2 cells [35]. Piegholdt et al. reported that the improvement of TJ integrity by treatment with isoflavone complex of biochanin A and prunetin is due to tyrosine phosphorylation of ZO-1 [36]. Therefore, TAE protected TJ function by inhibiting the increase in tyrosine phosphorylation of ZO-1 and by decreasing the interaction with ZO-1 and occludin in Caco-2 TNF-α-treated cells. 

To understand the underlying molecular mechanism by which TAE prevents TNF-α-induced intestinal epithelial barrier dysfunction, MLCK and NF-κB signals were studied. The results showed that TAE treatment attenuated TNF-α-induced disruption of the intestinal epithelial barrier function by inhibiting MLCK and NF-κB p65 protein levels (Figure 3). Previous studies have reported that MLC phosphorylation, which is activated by MLCK, generates TJ deterioration, and MLCK pathway activation is involved in NF-κB activation [13,37]. The translocation of the NF-κB subunit from the cytosol to the nucleus accelerates the synthesis of MLCK at the transcriptional level [13,19,37,38]. Therefore, we concluded that TNF-α disrupted TJ integrity by increasing MLCK expression through NF-κB activation; however, TAE inhibited TNF-α’s action, thus maintaining TJ integrity [5].

## 5. Conclusions

This study demonstrated that thinned apple, an indispensable agro-waste produced in apple cultivation, prevented the dysfunction of TJs in Caco-2 cells exposed to TNF-α. The preventive effect of TAE on the small intestinal epithelial barrier was superior to that of MAE. This may be due to the higher polyphenol content of TAE than that of MAE. Chlorogenic acid was the predominant polyphenol in TAE and MAE. Both TAE and MAE significantly inhibited TNF-α-induced inflammatory responses, such as IL-1 and IL-6 expression. Suppression of TNF-α-induced TJ dysfunction by MAE was found to be associated with increased expression of TJ proteins, such as claudin-1, -4, -5, ZO-1, and occludin. Moreover, TAE prevented TNF-α-induced ZO-1 phosphorylation and inhibited the ZO-1-occludin complex. TAE attenuated TNF-α-induced TJ deterioration via the NF-κB/MLCK pathway. In conclusion, thinned apples are a potential functional ingredient that can prevent intestinal epithelial dysfunction and inflammation.

## Figures and Tables

**Table 1:** Primer sequence.

Gene	Orientation	Primers Sequence (5′→3′)	NCBI Accession NO.
COX-2	Forward	CAAATCCTTGCTGT TCCCACCCAT	NM_000963.4
	Reverse	GTGCACTGTGTTTGGAGTGGGTTT	
IL-6	Forward	GGTACATCCTCGACGGCATCT	NM_001371096.1
	Reverse	GTGCCTCTTTGCTGCTTTCAC	
TNF-α	Forward	CCCAGGCAGTCAGATCATCTTC	NM_000594.4
	Reverse	AGCTGCCCCTCAGCTTGA	
IL-1β	Forward	TGGCAATGAGGATGACTTGTTC	NM_000576.3
	Reverse	CTGTAGTGGTGGTCGGAGATT	
Claudin-3	Forward	AAGGTGTACGACTCGCTGCT	NM_001306.4
	Reverse	AGTCCCGGATAATGGTGTTG	
Claudin-5	Forward	CTCTGCTGGTTCGCCAACAT	NM_003277.4
	Reverse	CACAGACGGGTCGTAAAACTC	
18S rRNA	Forward	GATGGAAAATACAGCCAGGTCCTA	NM_022551.3
	Reverse	TTCTTCAGTCGCTCCAGGTCTT	

**Table 2:** Polyphenol contents of thinned and mature apple extracts.

Total Polyphenols	Thinned Apple Extract (µg/g)	Mature Apple Extract (µg/g)
Chlorogenic acid	6172.03	965.02 ***
Epicatechin	2027.13	171.18 ***
Catechin	1646.39	380.81 ***
Phloridizin	1081.61	260.99 ***
Procyanidin B1	792.32	320.41 ***

The values are the means ± SD (*n* = 3). Significantly different between TAE and MAE by Student’s *t*-test at *** *p* < 0.001.

## Data Availability

The data presented in this study are available on request from the corresponding author.

## References

[1] Lee C.G., Lee S.Y., Joo S.Y., Cho L.H., Park S.Y., Lee S.H., Oh K.C., Kim D.H. (2017). A Study on Agricultural by-products for Biomass-to-energy Conversion and Korean Collecting Model. New Renew. Energy.

[2] Bhatia L., Johri S., Ahmad R. (2012). An economic and ecological perspective of ethanol production from renewable agro waste: A review. AMB Express.

[3] Rana S., Bhushan S. (2016). Apple phenolics as nutraceuticals: Assessment, analysis and application. J. Food Sci. Technol..

[4] Shoji T., Akazome Y., Kanda T., Ikeda M. (2004). The toxicology and safety of apple polyphenol extract. Food Chem. Toxicol..

[5] Boyer J., Liu R.H. (2004). Apple phytochemicals and their health benefits. Nutr. J..

[6] Gerhauser C. (2008). Cancer Chemopreventive Potential of Apples, Apple Juice, and Apple Components. Planta Med..

[7] Odenwald M.A., Turner J.R. (2017). The intestinal epithelial barrier: A therapeutic target?. Nat. Rev. Gastroenterol. Hepatol..

[8] Schneeberger E.E., Lynch R.D. (2004). The tight junction: A multifunctional complex. Am. J. Physiol. Physiol..

[9] Lee B., Moon K.M., Kim C.Y. (2018). Tight Junction in the Intestinal Epithelium: Its Association with Diseases and Regulation by Phytochemicals. J. Immunol. Res..

[10] Baumgart D.C., Carding S. (2007). Inflammatory bowel disease: Cause and immunobiology. Lancet.

[11] Al-Sadi R., Boivin M., Ma T. (2009). Mechanism of cytokine modulation of epithelial tight junction barrier. Front. Biosci..

[12] Kim C.Y. (2016). Inhibition of interleukin-1α-induced intestinal epithelial tight junction permeability by curcumin treatment in Caco-2 Cells in Caco-2 cells. J. Life Sci.

[13] Cao M., Wang P., Sun C., He W., Wang F. (2013). Amelioration of IFN-γ and TNF-α-induced intestinal epithelial barrier dysfunction by berberine via suppression of MLCK-MLC phosphorylation signaling pathway. PLoS ONE.

[14] Li N., Gu L., Qu L., Gong J., Li Q., Zhu W., Li J. (2010). Berberine attenuates pro-inflammatory cytokine-induced tight junction disruption in an in vitro model of intestinal epithelial cells. Eur. J. Pharm. Sci..

[15] Suzuki T., Tanabe S., Hara H. (2011). Kaempferol Enhances Intestinal Barrier Function through the Cytoskeletal Association and Expression of Tight Junction Proteins in Caco-2 Cells. J. Nutr..

[16] Suzuki T., Hara H. (2009). Quercetin Enhances Intestinal Barrier Function through the Assembly of Zonnula Occludens-2, Occludin, and Claudin-1 and the Expression of Claudin-4 in Caco-2 Cells. J. Nutr..

[17] Günzel D., Yu A.S.L. (2013). Claudins and the Modulation of Tight Junction Permeability. Physiol. Rev..

[18] Schwarz B.T., Wang F., Shen L., Clayburgh D., Su L., Wang Y., Fu Y.-X., Turner J.R. (2007). LIGHT Signals Directly to Intestinal Epithelia to Cause Barrier Dysfunction via Cytoskeletal and Endocytic Mechanisms. Gastroenterology.

[19] Amasheh M., Fromm A., Krug S.M., Amasheh S., Andres S., Zeitz M., Fromm M., Schulzke J.D. (2010). TNFα-induced and berberine-antagonized tight junction barrier impairment via tyrosine kinase, Akt and NFκB signaling. J. Cell Sci..

[20] Vreeburg R.A., E van Wezel E., Ocaña-Calahorro F., Mes J.J. (2012). Apple extract induces increased epithelial resistance and claudin 4 expression in Caco-2 cells. J. Sci. Food Agric..

[21] Finotti E., Gezzi R., Nobili F., Garaguso I., Friedman M. (2015). Effect of apple, baobab, red-chicory, and pear extracts on cellular energy expenditure and morphology of a Caco-2 cells using transepithelial electrical resistance (TEER) and scanning electron microscopy (SEM). RSC Adv..

[22] Wong X., Carrasco-Pozo C., Escobar E., Navarrete P., Blachier F., Andriamihaja M., Lan A., Tome A., Cires M.J., Pastene E. (2016). Deleterious effect of p-cresol on human colonic epithelial cells prevented by proanthocyanidin-containing polyphenol extracts from fruits and proanthocyanidin bacterial metabolites. J. Agric. Food Chem..

[23] Wang S., Li Q., Zang Y., Zhao Y., Liu N., Wang Y., Xu X., Liu L., Mei Q. (2017). Apple Polysaccharide inhibits microbial dysbiosis and chronic inflammation and modulates gut permeability in HFD-fed rats. Int. J. Biol. Macromol..

[24] Lauren D.R., Smith W.A., Adaim A., Cooney J.M., Wibisono R., Jensen D.J., Zhang J., Skinner M.A. (2009). Chemical composition and in vitro anti-inflammatory activity of apple phenolic extracts and of their sub-fractions. Int J Food Sci Nutr..

[25] Yoshioka Y., Akiyama H., Nakano M., Shoji T., Kanda T., Ohtake Y., Takita T., Matsuda R., Maitani T. (2008). Orally administered apple procyanidins protect against experimental inflammatory bowel disease in mice. Int. Immunopharmacol..

[26] Skyberg J.A., Robison A., Golden S., Rollins M.F., Callis G., Huarte E., Kochetkova I., Jutila M.A., Pascual D.W. (2011). Apple polyphenols require T cells to ameliorate dextran sulfate sodium-induced colitis and dampen proinflammatory cytokine expression. J. Leukoc. Biol..

[27] Zheng H.-Z., Kim Y.-I., Chung S.-K. (2012). A profile of physicochemical and antioxidant changes during fruit growth for the utilisation of unripe apples. Food Chem..

[28] Zhang P., Jiao H., Wang C., Lin Y., You S. (2019). Chlorogenic Acid Ameliorates Colitis and Alters Colonic Microbiota in a Mouse Model of Dextran Sulfate Sodium-Induced Colitis. Front. Physiol..

[29] Akazome Y., Kametani N., Kanda T., Shimasaki H., Kobayashi S. (2010). Evaluation of Safety of Excessive Intake and Efficacy of Long-term Intake of Beverages Containing Apple Polyphenols. J. Oleo Sci..

[30] Miura D., Miura Y., Yagasaki K. (2007). Effect of Apple Polyphenol Extract on Hepatoma Proliferation and Invasion in Culture and on Tumor Growth, Metastasis, and Abnormal Lipoprotein Profiles in Hepatoma-Bearing Rats. Biosci. Biotechnol. Biochem..

[31] Barmeyer C., Erko I., Awad K., Fromm A., Bojarski C., Meissner S., Loddenkemper C., Kerick M., Siegmund B., Fromm M. (2017). Epithelial barrier dysfunction in lymphocytic colitis through cytokine-dependent internalization of claudin-5 and -8. J. Gastroenterol..

[32] Watari A., Sakamoto Y., Hisaie K., Iwamoto K., Fueta M., Yagi K., Kondoh M. (2017). Rebeccamycin Attenuates TNF-α-Induced Intestinal Epithelial Barrier Dysfunction by Inhibiting Myosin Light Chain Kinase Production. Cell. Physiol. Biochem..

[33] Zhang Y., Ding X., Miao C., Chen J. (2019). Propofol attenuated TNF-α-modulated occludin expression by inhibiting Hif-1α/ VEGF/ VEGFR-2/ ERK signaling pathway in hCMEC/D3 cells. BMC Anesthesiol..

[34] González-Mariscal L., Tapia R., Chamorro D. (2008). Crosstalk of tight junction components with signaling pathways. Biochim. Biophys. Acta (BBA)-Biomembr..

[35] Kawauchiya T., Takumi T., Kudo Y., Takamori A., Sasagawa T., Takahashi K., Kikuchi H. (2011). Correlation between the destruction of tight junction by patulin treatment and increase of phosphorylation of ZO-1 in Caco-2 human colon cancer cells. Toxicol. Lett..

[36] Piegholdt S., Pallauf K., Esatbeyoglu T., Speck N., Reiss K., Ruddigkeit L., Stocker A., Huebbe P., Rimbach G. (2014). Biochanin A and prunetin improve epithelial barrier function in intestinal CaCo-2 cells via downregulation of ERK, NF-κB, and tyrosine phosphorylation. Free Radic. Biol. Med..

[37] Ma T.Y., Boivin M.A., Ye D., Pedram A., Said H.M. (2005). Mechanism of TNF-α modulation of Caco-2 intestinal epithelial tight junction barrier: Role of myosin light-chain kinase protein expression. Am. J. Physiol. Liver Physiol..

[38] Wang G., Sun G., Wang Y., Yu P., Wang X., Zhou B., Zhu H. (2019). Glabridin attenuates endothelial dysfunction and permeability, possibly via the MLCK/p MLC signaling pathway. Exp. Ther. Med..

